# The risk of an incident hospital contact with a musculoskeletal disorder in Danish occupational fishers: a register-based study

**DOI:** 10.1186/s12891-023-06274-4

**Published:** 2023-03-06

**Authors:** Line Nørgaard Remmen, David Høyrup Christiansen, Kimmo Herttua, Heidi Klakk, Gabriele Berg-Beckhoff

**Affiliations:** 1 Department of Public Health, Research Unit for Health Promotion, University of Southern Denmark, Esbjerg, Denmark; 2 Research Unit of Applied Health Science (SUPRA), University College South Denmark (UCSYD), Esbjerg, Denmark; 3 Department of Occupational Medicine, University Research Clinic, Danish Ramazzini Centre Goedstrup Hospital, Herning, Denmark; 4 Department of Clinical Medicine, Aarhus University, Health, Aarhus, Denmark; 5 Center for Health and Nursing Research, Regional Hospital Central Jutland, Research, Viborg, Denmark; 6 Elective Surgery Centre, Silkeborg Regional Hospital, Silkeborg, Denmark; 7 Department of Public Health, Center for Maritime Health and Society, University of Southern Demark, Esbjerg, Denmark; 8 Research Unit for Exercise Epidemiology (EXE), Department of Sports Science and Clinical Biomechanics (IOB), University of Southern Denmark, Odense M, Denmark; 9 Center for Clinical Research and Prevention, Section for Health Promotion and Prevention, The Capital Region, Frederiksberg, Denmark; 10 Hospital South West Jutland, University Hospital of Southern Denmark, Esbjerg, Denmark

**Keywords:** Occupational epidemiology, Occupational health, Register-based study, Musculoskeletal disorder, Occupational fishers, Back disorders

## Abstract

**Background:**

The prevalence of musculoskeletal disorders (MSDs) among occupational fishers is high, yet knowledge of the risk factors is scarce and inconsistent. The aim of this study was to investigate the risk from various work-related characteristics on incident hospital contact due to a musculoskeletal disorders and other pain disorders among Danish occupational fishers.

**Methods:**

This register-based study comprised data from the Danish Occupational Cohort with eXposure (DOC*X) for all persons registered as occupational fishers between 1994 and 2017. Time-to-event analysis with Cox regression model was used with age as the time scale.

**Results:**

Among the 15,739 fishers, 40% (n = 5,669 cases) had an incident hospital contact with an MSD during follow-up. Back disorders were the dominant complaint. Male fishers working less than 5 years or more than 15 years had higher risks of MSD (HR 2.40 (95% CI: 2.06, 2.80), HR: 2.04 (95% CI: 1.76, 2.35), respectively, than those working for over 20 years. Period effects confounded and reduced the risk from occupational seniority.

**Conclusion:**

Fishers occupational seniority vary in risk of MSDs across working life. Results showed a nonlinear relationship between the highest risk for fishers working less than 5 years and the lowest risk working more than 20 years as occupational fisher. More years in the workforce, a captain education, and primarily working part time significantly reduced the risk of experiencing a first MSDs for men. Healthy worker effect was documented.

**Supplementary Information:**

The online version contains supplementary material available at 10.1186/s12891-023-06274-4.

## Background

Musculoskeletal disorders (MSDs) are consistent contributors to the global burden of disease. The years lived with disability are particularly impacted by MSDs, which currently affect 1.7 billion people worldwide [1, 2]. MSDs are induced or aggravated by work and the circumstances of its performance. Many musculoskeletal complaints can become chronic and occur after exposure to work-related risk factors over a period of time [3]. As a consequence, MSDs are considered to be common cause of severe long-term pain and physical disabilities, both of which affect many aspects daily life [3]. MSDs also pose considerable economic burden to society due to sickness absence, interactions with the health care system, and early withdrawal from the labor market [1, 2].

One occupational area that is at high risk for MSDs is occupational fishery. Fishers carry out various tasks involving the manual handling (lift, pull and push) of heavy gear. They perform these tasks despite constant ship movements and vibrations [4, 5], often in wet and slippery surfaces, with long hours with limited opportunities for rest [6]. To address these problems, preventive musculoskeletal health initiatives have introduced new working methods, assistive devices, and techniques [7–9], but new international legislation on fishing quotas has put a strain on the workload demands within the industry [10]. Therefore, even with these preventive measures, occupational fishers are consistently being affected by MSDs [11, 12]. The prevalence of MSDs in occupational fishers worldwide is high, regardless of international differences in fishing methods, and ranges from 15 to 93%, depending on case definitions such as subjective MSD pain, self-reported-, or register-based MSD cases [7, 11].

Despite the high prevalence of MSDs among occupational fishers, little is known about the work-related risk factors in fishery or their impact on MSDs. Previous studies have investigated the work-related risk factors among occupational fishers but have yielded few clear conclusions. The sparse research is heterogenic in methodology and comprises studies of fluctuating quality ratings. One study suggested that working part time in fishery significantly increased the risk of MSDs [13]. However, inconclusive findings were seen for vessel type, job type, and occupational seniority [11, 14–17].

The occupational characteristics of fishers can vary widely as do the patterns of work duration for individual fishers. Fishing conditions change over seasons and time, and the job demands, and vessel types also vary. An occupational fisher may change fishing methods or shift to another trade multiple times over short- (season) and long-term (calendar years) periods [12, 13]. One important factor that has influenced the shifts between and within occupational fishery is the reform of fishing-quota legislation introduced in 2002. This legislation negatively affected the mental and physical health of occupational fishers because it resulted in increased physical workload, smaller crew sizes, and economic pressure [18]. However, no studies have investigated whether multiple shifts between fishery trades have had any impact on MSDs.

The heavy work and frequent manual of catch (lift, push and pull) cause considerable strain on the musculoskeletal system and will never be completely eliminated from fishery [19]. Therefore, we need to understand how specific characteristics of occupational fishing contribute to the development of MSDs. Several aspects need to be considered when analyzing the exposure from working conditions within occupational fishery on the risk of MSDs, as the individual risk is also affected by changing conditions within the labor market [20]. The introduction of fishing quota legislation in 2002 could be a period effect that changed the risk for MSDs [20]. Thus, the aim of this study was to investigate associations between various work-related characteristics and incident hospital contact due to MSDs and other pain disorders in occupational fishers. Moreover, we investigated the period effect related to the introduction of fishing quota legislation in 2002.

## Methods

### Data sources

This study was based on the Danish Occupational Cohort with eXposure (DOC*X), which contains information from well-established nation-wide registers [21], including those on labor market affiliation [22], and the Danish National Patient Registry [23]. Danish registers make it possible to link information on an individual level across all registers through a personal identification number with no loss of follow-up [24]. DOC*X provides employment status and occupational classification based on annual registration of all persons from the age 16 and is derived from the Employment Classification Module [21, 25].

### Study population

Several sources were used to define the occupational group to ensure good coverage. The study population of Danish occupational fishers was derived by including all Danish citizens who have ever been registered as fishery workers, for any duration, in The Danish version of the International Standard Classification of Occupation (DISCO) or who were registered as having a main affiliation with fishery within the industry classification (Supplementary Table 1) [26].

### Outcome variable

The incident hospital-registered MSD was the outcome of interest. Cases were identified through hospital records in the Danish National Patient Registry [23]. The included diagnoses are based on the Danish International Classification of Diseases version 10 (ICD-10). Persons with A (primary) or B (secondary) diagnosis for any MSD (M00-M99) and other pain disorders (G43* (migraine), G44* (nerve-related headache), G546 + G547 (phantom pain), G500A + G501 (fascial pain), G55* + G56* + G57* (nerve compression from discus/stenosis or in UE/LE), G89* (pain not elsewhere classified), R51* (headache) and R52*( nonspecific pain syndrome)) were chosen based on a previous study on MSD [27]. The MSD outcome included overall MSDs (M00-M99*) for any disorders followed by a categorization of diagnosis groups, presenting the most dominant groups by ICD-10 categorization. Information on injuries (ICD10: DS*) and accidents was excluded from our study.

### Exposure variables

We considered occupational seniority as the primary exposure. Methodologically, we aimed to treat the risk from fishery as an accumulation of exposure according to the length of affiliation [28]. Occupational seniority was thus expressed as cumulative years in the fishery trade and was further categorized into the following groups: greater than 20 years in the occupation (used as a reference in the analysis), 15 to < 20 years, 10 to < 15 years, 5 to < 10 years, and less than 5 years.

We also investigated several aspects within occupational fishery that could affect the risk of an MSD. As occupational fishery often has working patterns that involve shifting trades, we calculated the total number of shifts between trades over the years in the workforce. Thus, if a fisher transferred back to fishery multiple times from another trade, all transfers would be counted as a shift. Within the educational classification system, we registered those who had completed a formal education as captain to address the difference of work characteristics from that of the deckhands. From the Employment Classification Module, we calculated the percentage of life insured as being self-employed, and based on the Labour market status Register, we determined whether the fishery workers had primarily been insured as a full time or part time fisher throughout their years within the fishery trade.

### Covariates and confounder selection

**Fig. 1:**
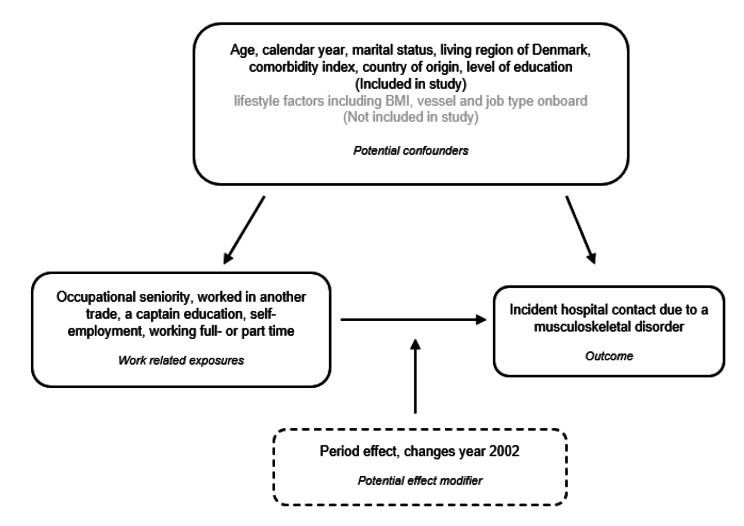
Illustration of the association between fishery work and risk of incident musculoskeletal disorder

To control for potential confounding, we considered the variables illustrated in Fig. 1. These variables were selected a priori based on available external validation according to the theory of Directed Acyclic Graphs (DAGs) [29, 30]. Figure 1 is an illustration inspired by the DAGs that reflects our assumptions about the causality paths from which the occupational fishery work affects the occurrence of an incident MSD and how these paths are affected in the presence of confounding conditions. The ‘open back-door’ criterion was used in selecting the included covariates to reduce the impact of confounding on the association [30]. A ‘backdoor path’ is an alternate path between exposure and outcome, where confounding is defined with at least one open back-door path [31]. The covariates included in the analysis were age, calendar year, region, gender, marital status, comorbidity.

### Statistical methods

Cox regression models were used to estimate crude (Model 1) and adjusted hazard ratios (HR) (Model 2) and the corresponding 95% confidence interval (95%CI) in Stata: Release 17 (StataCorp. 2021). After a crude model only adjusted for age as time scale, we adjusted analysis for overall years in the workforce, marital status, primary living region, country of origin, highest educational level and if the fishermen had any comorbidities within the time period. Comorbidity was calculated by the Charlson comorbidity index version 2000 [32]. The proportional hazard assumption was fulfilled and investigated using graphical visualization and the numerical approach [33, 34]. Age was used as the underlying time scale, which is suggested for longitudinal studies [35, 36]. Age as the time scale because we expected the hazard to be a function of age rather than time-in-study on the risk of MSD [36]. The computer time zeroed are birth, meaning that time is represented in terms of how far away event is from the date of birth. The time that fishers become at risk was set at the age they entered the workforce, given that they had lived to this age without having had a registered MSD.

To test for period effect, we looked into differences of data before and after the year 2002, the year when the reform of quota legislation was introduced, to address the possible changes of risk for MSDs. We investigated the period effect in two steps. First, we split our dataset based on the year 2002 to create two possible observations of risk sets per occupational fisher. We then conducted an adjusted Cox regression that included a test of the interaction between the two time periods and the work-related exposures of interest. Second (Model 3), we included period effect as a covariate in the adjusted model to investigate the difference of adjusted hazards from Model 2.

The follow-up period began in January 1994, and then annually included persons the year of their first registration to any employment in Denmark until 2017. Follow-up time was calculated in person-years as the difference between birth until the incident MSDs diagnosis or censoring by emigration (with a duration of over 5 years), death, or end of follow-up on December 1, 2017, whichever occurred first. Information on death and emigration were extracted from the Danish Civil Registration system [24].

## Results

*Sample characteristics*. A total of 15,739 persons were included in the study (Table 1). Great differences could be seen between the genders regarding work characteristics: the average years in the fishery occupation for males (17 years) was four times that of females and 10% of the male fishers, but none of the female fishers, were captains. Years in fishery ranged from 1 to 24, and most males had been in the trade for over 15 years. In contrast, most of the female fishers had been in the trade for less than 5 years. Regardless of gender, there was an average of five shifts between trades during the years in the workforce. Fishers predominantly worked full time, and most were not self-employed.

**Table 1:** Demographic and work-related characteristics of the included 15,739 Danish occupational fishers from the Danish Occupational Cohort (DOC*X) registered in the years of 1994–2017 (n = 15,739)

Demographic characteristics		Male fishers (n = 13,165)		Female fishers (n = 2574)	
Age entering follow-up, average (SD)		44.58	16.10	26.62	8.61
Age at end of follow-up, average (SD)		63.99	13.76	49.43	20.62
Primary marital status n (%)	Married	6966	52.90	946	36.75
	Other	6199	47.09	1628	63.25
Educational level, n (%)	Short mandatory	9918	75.34	352	13.68
	Higher	3247	24.66	2222	86.32
Captain education, n (%)	Yes	1353	10.28		
Land of origin, n (%)	Denmark	12,872	97.77	2440	94.79
Region of Denmark*, n (%)	Northern Jutland	3786	28.76	281	10.92
	Central Jutland	3204	24.34	576	22.38
	Southern Jutland	2778	21.10	528	20.51
	Capital area	2061	15.66	810	31.47
	Zealand	1317	10.00	379	14.72
**Work characteristics**					
Years in cohort, average (SD)		23.97	0.48	23.98	0,23
Years as fisherman, average (SD)		16.86	6.86	3.95	4.12
Years as fisherman, n (%)	> 20 years	5232	39.74	28	1.09
	16-<20 years	3578	27.18	75	2.91
	11-<15 years	1595	12.12	100	3.88
	5-<10 years	1397	10.61	228	8.86
	< 5 years	1363	10.35	2143	83.26
Occupational shifts, average (SD)		5.42	3.95	5.05	2.84
Self-employed during working life (%)	min/50%	11,321	85.99	2553	99.18
	50/max%	1844	14.01	21	0.82
Full- or parttime work, n (%)	Full time	11,867	90.14	2398	93.16
	Part time	1298	9.86	176	6.84

Abbreviations: SD (standard deviation), n (number)

Blanks are due to low number of observation n < 5

*Events of musculoskeletal disorders.* Approximately 40% of the occupational fishers experienced an incident MSD (M00-M99) within the follow-up period (Supplementary Table 2). The most dominant MSD group was “Soft tissue disorders.” The most incident specific complaint was “Back disorders”. Back disorders accounted for more than a fourth of male fishers, while it accounted for one-fifth of female fishers.

*Adjusted cumulative hazards of overall MSD.* Analyses were stratified by gender to handle the observed significant interaction effect (Table 2). **For males**, the analysis showed that, compared to those who had worked more than 20 years within the fishery trade, the fishers working less than 5 years had a significantly higher risk of MSD (HR 2.40 (95%CI: 2.06, 2.80)) when adjusting for years in any workforce, marital status, region, comorbidity, country of origin, and educational level. Years in any workforce accounted for most of the observed confounding effect. Comparison of the remaining categories of fishery occupational seniority revealed that any number of years working in fishery showed significantly increased risk for MSD, when compared to working more than 20 years in the trade. Adjusted analysis showed that working more than the average number of years in any workforce significantly reduced the risk of MSD (HR 0.17 (95%CI:0.16, 0.18)). Likewise, adjusted analysis showed that having a captain education and mainly working part time were associated with a lower risk, HR 0.79 (95%CI:0.72, 0.86) and HR 0.71 (95%CI: 0.65, 0.78), respectively. More than five shifts of trade significantly increased the risk (HR 1.17 (95% CI: 1.09, 1.25)) of experiencing an MSDs. No significant association was seen between the first incident of MSD and self-employment. An analysis of age cohort showed that, men who entered the cohort at age 16 had significantly reduced risk of MSDs HR 0.53 (95% CI:0.45,062) compared to those born before 1976.

**Table 2:** Hazard ratios from the occupational fishery work exposures on incident hospital contact with any musculoskeletal disorders between 1994 and 2017 (n = 15,739)

		Model 1		Model 2		Model 3	
		Person-years: 89,906,617				Person-years: 82,239,890	
Occupational fishery exposures **for men**:		HR	95%CI	HR	95%CI	HR	95%CI
Binary age (age cohort)	Ref; born before 1976	**0.62**	**0.54, 0.71**	**0.18**	**0.14, 0.22**	na	na
Years in workforce^1^	Ref; <average 15.73	**0.17**	**0.16, 0.18**	**0.17**	**0.16, 0.18**	**0.82**	**0.78, 0.88**
Years as fisherman	> 20 years	1	ref	1	ref	1	Ref
	16-<20 years	**2.63**	**2.29, 3.04**	**2.04**	**1.76, 2.35**	**1.89**	**1.59, 2.24**
	11-<15 years	**4,63**	**4.05, 5.30**	**1.94**	**1.69, 2.24**	**1.22**	**1.02, 1.46**
	5-<10 years	**7.08**	**6.62, 8.07**	**1.71**	**1.48, 1.98**	0.95	0.78, 1.14
	< 5 years	**15.05**	**13.03, 17.12**	**2.40**	**2.06, 2.80**	**2.83**	**2.33, 3.45**
N = occupational shifts	Ref < average 3.19	**0.38**	**0.36, 0.41**	**1.17**	**1.09, 1.25**	1.06	0.98, 1.15
Captain education	Ref; no	1.06	0.97, 1.15	**0.79**	**0.72, 0.86**	**0.82**	**0.74, 0.90**
Self-employed during working life (%)	Ref; min/50	**0.39**	**0.36, 0.43**	1.01	0.92, 1.11	**1.10**	**1.03, 1.17**
Full- or part time work^2^	Ref; Full time	**1.13**	**1.03, 1.23**	**0.71**	**0.65, 0.78**	1.09	0.90, 1.30
Occupational fishery exposures **for women**:		HR	95%CI	HR	95%CI	HR	95%CI
Binary age (age cohort)	Ref; born before 1976	0.91	0.72, 1.14	1.06	0.84, 1.34	na	na
Years in workforce^1^	Ref; < average 21.61	**0.25**	**0.23, 0.28**	**0.25**	**0.22, 0.28**	**0.67**,	**0.61, 0.76**
Years as fisherman	> 5 years	1	Ref	1	Ref	1	Ref
	< 5 years	**1.62**	**1.35, 1.95**	**1.69**	**1.40, 2.05**	**1.86**	**1.45, 2.37**
N = occupational shifts	Ref; < average 7.46	**0.57**	**0.51, 0.65**	**0.81**	**0.71, 0.93**	**0.78**	**0.67, 0.90**
Self-employed during working life (%)	Ref; min/50	0.56	0.30, 1.07	0.88	0.46, 1.68	1.03	0.90, 1.17
Full-or part time work^2^	Ref; Full time	0.93	0.74, 1.16	0.99	0.79, 1.24	1.07	0.74, 1.56

Abbreviations: HR (Hazard ratio), CI (confidence interval), ref (statistical reference), na (not applicable)

^1^ Years in workforce until event or censoring

^2^ Presented as the mode of possible variable categories throughout their follow-up period

**Bold**: significant associations

Model 1: Crude model, age as time scale

Model 2: Adjusted for: years in workforce, marital status, region, Charlson 2000 comorbidity index, country of origin, educational level, age as time scale

Model 3: Adjusted for abovementioned covariates and adding period (before and after 2002), age as time scale

**For females**, we found fewer significant associations between occupational characteristics and MSDs. Working more than 5 years compared to less than 5 years significantly increased the risk for MSDs (HR 1.62 (95%CI:1.35, 1.95)) when adjusting for all covariates. More than the average number of shifts and years in workforce significantly reduced the HR of experiencing an MSDs.

*The period effect*. Investigation of the impact of the new quota legislation introduced in 2002 by making dummy variables, splitting for the year 2002, showed no significant interaction (Table 2). In Model 3, we added period as a covariate to capture the period effect before and after this quota legislation reform. For men, adjusting for the period in most exposures decreased the associations seen in Model 2, showing significantly increased HR of years in workforce, working < 5 years in fishery (HR:2.83 95% CI:2.33,3.45), self-employment, and having a captain education. Associations for working less than between 10 and 20 years significantly increased the risk when adjusting for the effect of this period.

*Adjusted cumulative hazards of specific MSDs*. Occupational seniority of under 20 years increased risk for back, knee, shoulder, and “other pain” disorders. For both genders, the highest estimate of risk for experiencing any of the specific MSDs was when working less than 5 years (Table 3). For male fishers, more than five occupational shifts significantly increased the risk for shoulder lesions and other pain disorders but did not significantly affect the risk of back or knee disorders. Male fishers with a captain education had a significantly decreased risk of shoulder lesions and other pain disorders. Lastly, significant results showed that male fishers who worked part time had a reduced risk of knee and other pain disorders, HR: 0.69 (95% CI:0.54, 0.88) and HR:0.66 (95% CI: 0.52, 0.85), respectively. In female fishers, the highest risk from occupational seniority was seen for experiencing other pain disorders (HR: 3.31 (95% CI:1.84, 5.94)). Female fishers who shifted occupation (> 5 times) had a significantly decreased risk of experiencing back disorders, knee disorders, and shoulder lesions.

**Table 3:** Adjusted hazard ratios from the occupational fishery work exposures stratified for gender on incident outcome for specific disorders between 1994 and 2017 (n = 15,739)

		Back disorders		Knee disorders		Shoulder lesions		Other pain disorders	
		Person-years under observation: 89.906.617							
		Cases: 1.643		Cases: 1.018		Cases: 514		Cases: 1.035	
Occupational fishery exposures **for men**:		aHR1	(95%CI)	aHR	(95%CI)	aHR	(95%CI)	aHR	(95%CI)
Years as fisherman	> 20 years	1	ref	1	Ref	1	Ref	1	Ref
	15-<20 years	**1.57**	**1.18, 2.09**	**2.68**	**1.82, 3.94**	**2.63**	**1.52, 4.57**	**1.99**	**1.38, 2.88**
	10-<15 years	**1.37**	**1.03, 1.82**	**2.29**	**1.56, 3.36**	**3.64**	**2.14, 6.20**	**2.15**	**1.50, 3.09**
	5-<10 years	**1.38**	**1.03, 1.84**	**2.13**	**1.43, 3.16**	**2.85**	**1.64, 4.98**	**1.80**	**1.23, 2.63**
	< 5 years	**1.87**	**1.37, 2.54**	**2.83**	**1.86, 4.13**	**3.55**	**1.96, 6.42**	**2.58**	**1.73, 3.86**
Occupational shifts (n)	Reference < average 3.19	1.09	0.94, 1.26	1.01	0.84, 1.21	**1.30**	**1.02, 1.67**	**1.33**	**1.11, 1.59**
Captain education	Reference; no	0.85	0.68, 0.95	0.81	0.64, 1.02	**0.68**	**0.50, 0.92**	**0.73**	**0.58, 0.91**
Self-employed during working life (%)	Reference; min/50	0.86	0.70, 1.07	1.07	0.84, 1.36	0.86	0.61, 1.20	0.82	0.64, 1.07
Full- or part-time work	Reference; Full time	0.94	0.79, 1.13	**0.69**	**0.54, 0.88**	0.78	0.55, 1.11	**0.66**	**0.52, 0.85**
Occupational fishery exposures **for female fishers**:		aHR	95%CI	aHR	95%CI	aHR	(95%CI)	aHR	(95%CI)
Years as fisherman	> 5 years	1	Ref	1	Ref	1	Ref	1	Ref
	< 5 years	**1.56**	**1.01, 2.44**	1.32	0.79, 2.18	**2.49**	**1.11, 5.60**	**3.31**	**1.84, 5.94**
Occupational shifts (n)	Reference < average 7.46	**0.69**	**0.50, 0.96**	**0.64**	**0.44, 0.95**	**0.45**	**0.35, 0.79**	0.85	0.62, 1.16
Self-employed during working life (%)	Reference; min/50	0.66	0.09, 4.97	1.91	0.56, 6.51				
Full- or part-time work	Reference; Full time	0.78	0.43, 1.45	0.70	0.34, 1.45	0.97	0.42, 2.24	0.98	0.57, 1.70

Abbreviations: HR (Hazard ratio), CI (confidence interval), ref (statistical reference)

**Bold**: significant associations

Adjusted for: years in workforce, marital status, region, Charlson 2000 comorbidity index, country of origin, educational level with age as time scale

## Discussion

To our knowledge, this is the first study to investigate associations between work-related characteristics and incident hospital contacts with MSDs while concurrently addressing the effect of a historical period within the trade. Surprisingly, we found no association between occupational seniority and increased risk of MSDs with accumulating years, but rather a nonlinear relationship. Our results showed that both male and female fishers had the highest risk for MSD when working less than 5 years. In males, the association between occupational seniority and MSD was non-linear. Some of the decline in risk within the highest occupational seniority category (> 20 years) could be attributed to the healthy worker effect indicating that those who are the healthiest remain in the occupation [37].

We carried out a longitudinal cohort study with a 24-year time span and included time-varying covariates to address the changes during the individuals’ working lives. Only two studies, covering a 1-year [13] and 5-year span [12], have previously investigated longitudinal risk of fishery work and the occurrence of MSDs. Three studies investigated the occupational seniority numerically but found little or no significant associations [14, 15, 17]. A cross-sectional study from Egypt showed an OR of 1.07 for current MSD [14], indicating an inclining risk per year in the trade. That study did not, however, handle categories of occupational seniority, which could reflect the nonlinear changes of risk we found over the accumulation of years. Our study illustrates the importance of categorizing occupational seniority to see the time varying risk from fishery work on the risk of MSD, as the difference when accumulating years without categorization diminishes the nonlinear association we found.

Different work-related characteristics had different effects on MSDs risk estimates. The characteristics that lowered the risk of MSDs were more years in the workforce, a captain education, and working mostly part time during working life. In contrast, the risk of MSDs increased significantly when fishers shifted between trades. Years in workforce significantly reduced the risk of incident MSDs but did also have the greatest confounding effect for the association of the effect from years in fishery. The healthy worker effect may be a contributing cause of the lower incidence found when having more years in workforce – because “those who work are healthier” than those who stop working [37]. Having a captain education lowered the risk of MSDs. This is not surprising since a captain’s workload compared to a deckhand’s entails less manual lifting and more tasks related to planning and sales [13]. Both status on ship – the highest being a captain – and fishing experience caused differences in responsibilities and tasks, where the least experienced fishers performed the heaviest tasks [13]. Moreover, captains may also tend to work longer in the fishing trade as they are more economically tied to their ship and the trade. We found a significant decrease in the risk of MSDs in fishers working part time compared to those working full time. This is contrary to the reverse association seen in a North Carolina cohort study [13]. However, the North Carolina study presented results from one year, whereas our results were based on 24 years of follow-up. This difference in follow-up time may partly explain the difference in estimates along with differences in country-specific characteristics [13]. However, cautiousness is needed to determine causality. The fishers working part time could either have another job that enables them to have less, or withstand, more strain. A population selection factor or the healthy worker effect could have contributed to the findings concerning part-time fishers [13]. Shifts naturally exists within the occupation as fishing conditions and demands change over seasons and time [12, 13]. All in all, it is unclear whether this relationship is causal, as we cannot state if they shift jobs due to already experienced pain, or if they experience increased pain because of the shift.

Congruent with previous studies, our study revealed that fishers are most affected by back disorders [13, 15, 17, 38–40]. One cause of such a consistently high incidence of back disorders among fishers is the biomechanical load that is required to counteract ship motion. The lower extremities and low back, in particular, have an increased workload when fishers constantly have to adapt to the motions of the ship during heavy work involving work postures such as lifting, pulling and pushing [41]. For men, fishery characteristics differed in terms of the risk of specific MSD body areas, although all men were significantly at risk from years in the trade. Interestingly, the high incidence of back disorders in men showed no significant association with other occupational characteristics besides years in trade. This suggests that back disorders are not indicated by these specific characteristics but are a consequence of length within the trade regardless of any other factors. Our results showed highly significant associations between years in fishery and the risk of “other pain disorders” in both genders. Future studies should incorporate these disorders as part of MSD investigations and preventive measures, as the consequences have an equally negative impact on individual well-being and societal costs.

We investigated the effect of the massive reform of fishing-quota legislation in 2002, as we anticipated that this could have an impact on number of MSD incidences in fishers. Taking period effect into account, the analysis showed no significant difference between the periods before and after the year 2002. However, including the period as a covariate significantly reduced certain risk estimates. The fishery trade has developed continuously across three decades, which might explain the modest change seen in our result. Though, these results suggest that future research should further consider the period effect when investigating the accumulation of occupational seniority longitudinally.

We carried out our analysis with age as the underlying time-exponent as suggested by previous studies [35, 36]. Nevertheless, the picture of development of MSDs and their association with increased age is an important, but difficult task to fully grasp considering occupational seniority. A common problem with separating age, period, and cohort effects is the ‘identifiability problem’, whereby a specific variable can be perfectly predicted by a linear combination of the remaining variables, resulting in no unique set of regression parameters [42]. In our study, it was clear that major collinearity exists when investigating age and occupational seniority. The importance of age was supported by our binary age cohort investigation, where adjusted analysis showed that men who entered the cohort at the age of 18 had a significantly reduced risk of MSDs. Thus, that a higher age when entering cohort was associated with a greater risk of incident MSD aligns with the natural mechanism – that the higher age causes higher risk for MSD. Although age is not an independent risk factor for work-related MSD, older workers are more susceptible to the working conditions than are younger workers due to decreased functional capability and prolonged exposure [43, 44].

Some important methodological limitations should be kept in mind when interpreting our findings. Firstly, our study population was derived from multiple registers to ensure good coverage. As a result, we may have overestimated the selection of the study population and included more persons registered as fishers than seen in previous studies [10, 12]. Data from the Danish Fishery Agency shows a lower number of fishers with an A-registration (A-registered can be owner of ship and quota) compared to our study population. Our analysis consisted of annual registrations, whenever a person was registered, no matter their registration enabling them to own ships and quotas. However, we do not believe this changed the associations shown, as we categorized by seniority. Secondly, an important caution lies again in the completeness of our case detection. Our outcome measure exclusively consisted of ‘the worst’ MSDs cases, where the fishers’ experience of pain and disability were so severe that they needed to be examined in the health care system. That the data comprised the worst MSDs cases reflects our experience that fishers contact doctors less when in pain than those of the general population [18]. Thirdly, we did not investigate the influence of lifestyle (i.e. BMI, smoking, eating and sleep habits) as no data were available. Other studies have suggested that BMI has an impact on the occurrence of MSDs [45]. However, populations defined by occupation are often more homogenous in several characteristics, including lifestyle choices, where e.g., job exposure matrices have been used for population-based estimates [21]. We consider fishers to be somewhat homogenous in lifestyle traits to such a degree that the findings may not have been highly influenced by this. Furthermore, to consider partially the effect of unhealthy lifestyle the Charlson comorbidity index was included as a confounder. Lastly, no definitive inference can be made on the causality between the occupational fishers’ risk on MSD partly because we were not able to disentangle the collinearity between occupational seniority and age effect. The risk from several characteristics of occupational fishery coincide and add to the complexity of a causal interpretation.

## Conclusion

In conclusion, 40% of the 15,739 fishers experienced an incident hospital contact due to MSD within the follow-up period of 24 years. Of those, the most dominant cases for both genders were back disorders. The gender-stratified analysis showed that both female and male occupational fishers with less than 5 years in occupation had the highest risk of overall and specific MSD, whereas male fishers with the highest seniority level above 20 years in trade had the lowest risk of all types of MSD. Our study illustrates a non-linear relationship between the occupational seniority level of fishers and their risk of an incident hospital contact due to MSDs. Thus, our findings suggest that categorization of accumulated years in the occupation should be explored in future research. More years in the workforce, a captain education, and primarily working part time significantly reduced the risk of experiencing an incident MSD for men. A similar pattern was seen for female fishers. More shifts than average between occupational trades increased risk of MSD for men, while lowering the risk at female fishers. The effects of occupational seniority were primarily attenuated by the period effect of the new reform of quota legislations in 2002. Future studies entailing multiple time-varying exposures and repeated disorders are warranted to fully understand the mechanism behind the development of MSDs in occupational fishery.

## Electronic supplementary material

Below is the link to the electronic supplementary material.

Supplementary Material 1

## Data Availability

The data that support the findings of this study are derived from registers in Statistics Denmark with an application through the Danish Occupational Cohort with Exposure (https://doc-x.dk/). Data from Statistics Denmark are available for Danish researchers, thereto restriction apply to the availability of these data, which were used under license for the current study, and are therefore not publicly available. Please contact corresponding author (lremmen@health.sdu.dk) for any questions.

## Abbreviations

DAGs: Directed Acyclic Graphs
DOC*X: Danish Occupational Cohort with eXposure
HR: Hazard ratios
ICD-10: International Classification of Diseases version 10
MSDs: Musculoskeletal disorders
95%CI: 95% Confidence Interval
