# Supplementary material for: The risk of an incident hospital contact with a musculoskeletal disorder in Danish occupational fishers: a register-based study
Source: BMC Musculoskelet Disord. 2023 Mar 6;24:168. doi: 10.1186/s12891-023-06274-4 (PMC9987051; doi:10.1186/s12891-023-06274-4)
Supplement: Supplementary file 1 — Supplementary Material 1 [file 12891_2023_6274_MOESM1_ESM.docx]

**Supplementary Table 1:** Overview of categorization of occupational fishery employment from Employment Classification Module (Danish: AKM) in the period of 1994-2017 of Danish Occupational Fishers

| **Classification system** | **Year** | **Classification Version** | **Classification code** |
| --- | --- | --- | --- |
| Occupational classification | 1988-2008 | Annual labor market affiliation code DISCO-88 (The Danish version of the International Standard Classification of Occupation, ISCO) | 6152 or 6153 |
|  | 2008-2017 | Annual labor market affiliation code DISCO-08 (as above) | 6222 or 6223 |
| Industry classification | 1994-2000 | brchl (Industry classification code for paid employees) or brchi (Industry classification code for self-employed) | 13010 |
|  | 2000-2007 | nacea (Industry classification code for paid employees) or nacei (Industry classification code for self-employed) | 50100 |
|  | 2007-2017 | NACEA_DB07 (Industry classification code for paid employees) or NACEI_DB07 (Industry classification code for self-employed) | 3110 or 3120 |

**Supplementary Table 2**: Overview over first hospital (in and outpatient) contact with musculoskeletal disorder of occupational fishers between 1994 and 2017 (cases n=7,257).

|  | | | | **Overall**  **(15.739)** |  | **Men**  **(n=13.165)** | | **Female fishers**  **(n=2.574)** | |
| --- | --- | --- | --- | --- | --- | --- | --- | --- | --- |
| **Musculoskeletal disorder (MSD)** | | | | n | % of all fishers | n | % of all men | n | % of all women |
|  | Any MSD (M00-M99) | | | 6218 | 39.50 | 5215 | 39.61 | 1003 | 38.97 |
|  | Other pain disorders (^*1^) | | | 1035 | 6.58 | 826 | 6.27 | 209 | 8.12 |
| **Specific MSD:** | | | |  |  |  |  |  |  |
|  | Arthrosis (DM15*/DM19*) | | | 840 | 5.34 | 791 | 6.01 | 49 | 1.90 |
|  |  | Arthrosis of knee (DM17*) | | 423 | 2.69 | 404 | 3.07 | 19 | 0.74 |
|  |  | Arthrosis of hip (DM16*) | | 250 | 1.59 | 239 | 1.82 | 11 | 0,43 |
|  | Knee disorders (DM22*/DM23*) | | | 595 | 3,78 | 462 | 3.51 | 133 | 1.28 |
|  | Back disorders (M40*/DM54*) | | | 1643 | 10.44 | 1434 | 10.89 | 209 | 8.12 |
|  |  | Back pain (DM54*) | | 694 | 4.41 | 586 | 4.45 | 108 | 4.20 |
|  |  | Disc disorders (DM50*/DM51*) | | 591 | 3.76 | 541 | 4.11 | 50 | 1.94 |
|  | Soft tissue disorders (DM60*/DM79*) | | | 2147 | 13.64 | 1718 | 13.05 | 429 | 16.67 |
|  |  | Muscle strain (DM626) | | 223 | 1.42 | 147 | 1.12 | 76 | 2.95 |
|  |  | Ganglion (DM674) | | 122 | 0.78 | 90 | 0.68 | 32 | 1.24 |
|  |  | Fibromyalgia (DM72*) | | 195 | 1.22 | 181 | 1.37 | 14 | 0.54 |
|  |  |  | Dupuytren syndrome (DM720) | 169 | 1.07 | 169 | 1.28 |  |  |
|  |  | Shoulder lesions (DM75*) | | 514 | 3.27 | 427 | 3.24 | 87 | 3.38 |
|  |  |  | Rotator cuff syndrome (DM751*) | 131 | 0.83 | 110 | 0.84 | 21 | 0.82 |
|  |  | Myalgia (DM79*) | | 434 | 2.76 | 348 | 2.64 | 92 | 3.57 |
|  | Carpal tunnel syndrome (DG560*) | | | 259 | 1.65 | 218 | 1.66 | 41 | 1.59 |

^*1^ Included in “other pain disorders” are: DG43*, DG44*, DG546 DG547 DG500A DG501 DG55* DG56** DG57* DG89* DR51* DR52*.
